# Psychological distress as a putative host-state determinant of cancer immunotherapy response

**DOI:** 10.3389/fimmu.2026.1891642

**Published:** 2026-07-30

**Authors:** Agnieszka Bronisz, Klaudia Kiel, Jakub Godlewski

**Affiliations:** 1 Tumor Microenvironment Laboratory, Mossakowski Medical Research Institute, Polish Academy of Sciences, Warsaw, Poland; 2 Department of NeuroOncology, Mossakowski Medical Research Institute, Polish Academy of Sciences, Warsaw, Poland

**Keywords:** cancer immunotherapy, cortisol, host immune state, immune checkpoint inhibitors, neuroimmune signaling, psychological distress, psycho-neuro-immunology

## Abstract

Cancer immunotherapy has transformed oncology, yet durable benefits remain limited to a subset of patients, and those cases are incompletely explained by tumor-intrinsic biomarkers alone. Growing evidence indicates that the host immune state is a critical contextual determinant of therapeutic response and may itself be shaped by psychological distress. In cancer patients, such distress-related states have been associated with altered neuroendocrine activity and related cortisol regulation, inflammation, reduced natural killer cell function, and broader immune dysregulation. In parallel, preclinical studies show that glucocorticoid and adrenergic signaling can impair antitumor immunity by promoting T-cell dysfunction, metabolic exhaustion, and increased inhibitory signaling, as well as suppressing antigen presentation. Emerging clinical studies in patients receiving immune checkpoint inhibitors further suggest that emotional distress before immunotherapy initiation is associated with poorer progression-free survival, response, and overall survival outcomes in several cancer settings, including non-small-cell lung cancer, gastroesophageal cancer, gastric cancer, and recurrent high-grade glioma. As these patient data remain largely observational, current evidence supports psychological distress as a putative host-state modifier or correlate, rather than as a proven causal determinant, of immunotherapy response. These observations support a psycho-neuro-immune framework in which psychological distress may identify or contribute to a host state that is less permissive for effective immunotherapy. Here, we review evidence linking host immune competence to immunotherapy efficacy, summarize clinical and molecular data connecting distress to immune dysregulation in cancer, and incorporate emerging evidence that brain–body signaling may shape tumor immunity through neural, endocrine, and immune pathways. We propose that psychological distress should be considered not merely as a parallel quality-of-life variable, but as a biologically relevant and clinically verifiable host-state factor with implications for biomarker development and future host-modulation immunotherapy trials.

## 1 Introduction

Psychological distress is a plausible but underappreciated host-state modifier in cancer immunotherapy. Current understanding of cancer immunotherapy response remains dominated by tumor-intrinsic biomarkers, yet these markers explain only part of why some patients derive durable benefit, whereas others do not. Measures such as programmed death-ligand 1 (PD-L1) expression and tumor mutational burden have clinical value. Still, they cannot fully capture whether the host immune system is in a state that can be effectively mobilized by immune checkpoint blockade (1–5). This limitation has increased interest in host-side determinants of response, particularly those that reflect systemic immune competence rather than tumor features alone (1, 4, 5).

Cancer is increasingly understood not only as a disease of transformed cells, but also as a disease of altered host-tumor interaction. Systemic immunity, inflammatory tone, circulating immune-cell composition, and broader host physiology, including active crosstalk with the nervous system, all contribute to whether an antitumor immune response can be initiated, sustained, and therapeutically reinvigorated (1, 4–14). In this broader framework, the key clinical question is not simply whether a tumor harbors a targetable immune feature, but whether the patient’s immune system remains sufficiently coordinated, competent, and responsive to treatment.

In this review, psychological distress is used as an umbrella term rather than as a single psychiatric condition. Psychological distress should be distinguished from generalized stress, a broader biological and adaptive process encompassing the organismal response to real or perceived challenges to homeostasis. Conversely, psychological distress captures the subjective emotional and cognitive-affective burden associated with such challenges (15). It refers to patient-reported emotional and psychosocial states that may include depressive symptoms, anxiety symptoms, perceived stress, reduced emotional functioning, and low social support. Across the clinical literature reviewed here, these dimensions are captured using a range of instruments, including depression and anxiety scales, emotional functioning subscales, health-related quality-of-life questionnaires, and study-specific distress measures (16, 17). This distinction is also important as distress-related states are heterogeneous and may include anxiety linked to uncertainty, persistent negative affect, and repetitive cognitive processes such as rumination (18). Such heterogeneity is important: psychological distress should not be treated as a uniform exposure, and differences in instrument choice, assessment timing, and symptom domains may partly explain variation across studies. We distinguish psychological distress from related but non-equivalent clinical factors such as pain, fatigue, sleep disruption, neurologic symptoms, corticosteroid exposure, socioeconomic adversity, and advanced disease burden. These factors can contribute to distress and may share biological pathways with distress-related states, but they can also independently influence immune function and cancer outcomes. They should therefore be considered potential confounders, mediators, or effect modifiers rather than interchangeable measures of psychological distress. Throughout this review, we use “distress-related states” to refer collectively to these overlapping psychological constructs, and we reserve stronger causal language for settings where experimental or longitudinal evidence supports such an interpretation. Within this broad construct, distress-related states in cancer patients have been linked to altered cortisol regulation, inflammatory activation, impaired natural killer cell activity, and broader immune dysregulation (14, 19–28). These observations suggest that distress may not merely accompany cancer as a parallel psychosocial burden, but may instead help shape biological states relevant to treatment responsiveness.

This possibility is especially relevant to immunotherapy. Preclinical studies show that glucocorticoid and adrenergic signaling can impair antitumor immunity by promoting T-cell dysfunction, metabolic exhaustion, altered antigen presentation, and inhibitory tumor-immune signaling (29–33). Emerging clinical cohorts further suggest that emotional distress assessed before treatment initiation is associated with poorer outcomes in several cancers treated with immune checkpoint inhibitors (34–40). Taken together, these data justify evaluating distress not only as a psychosocial variable, but also as a candidate determinant of host immune readiness in immunotherapy.

**Figure 1:**
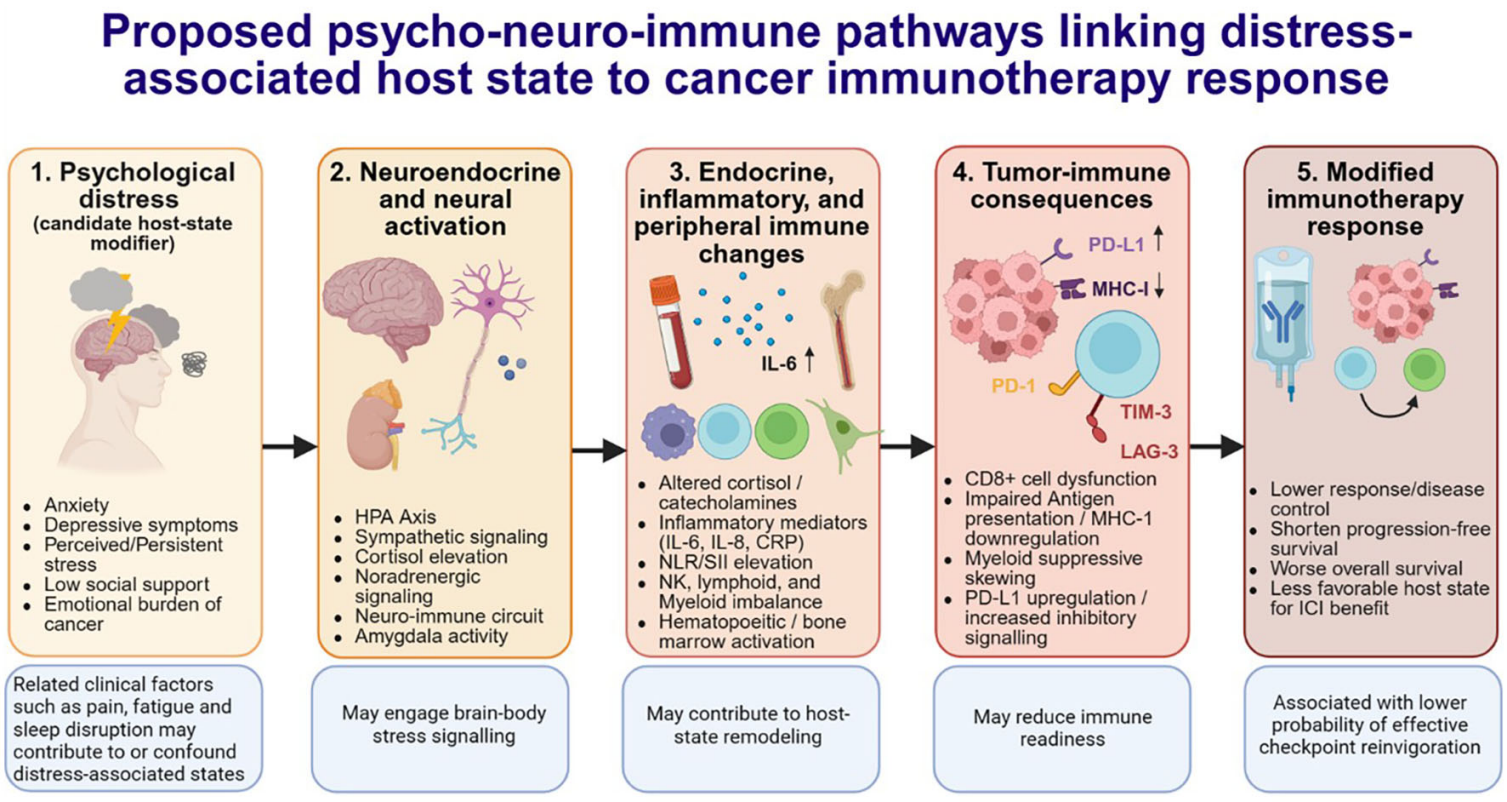
Psychological distress as a psycho-neuro-immune regulator of cancer immunotherapy response. Related clinical factors such as pain, fatigue, and sleep disruption may contribute to or confound distress-associated states (box 1), which, in turn, may influence the host immune state through neuroendocrine and neural pathways (box 2). These signals are associated with inflammatory remodeling and peripheral immune alterations, and may therefore contribute to host-state signaling (box 3). Preclinical studies further suggest that downstream effects on tumor-immune crosstalk (box 4) may define a host state that is less permissive to effective immune checkpoint blockade (box 5). Patient studies support a clinical association, whereas mechanistic links are derived mainly from preclinical models. HPA, hypothalamic-pituitary-adrenal; IL-6, interleukin-6; IL-8, interleukin-8; CRP, C-reactive protein; NLR, neutrophil-to-lymphocyte ratio; SII, systemic immune-inflammation index; NK, natural killer; PD-1, programmed cell death protein 1; TIM-3, T-cell immunoglobulin and mucin-domain containing-3; LAG-3, lymphocyte-activation gene 3; MHC-I, major histocompatibility complex class I; PD-L1, programmed death-ligand 1; ICI, immune checkpoint inhibitor. Upward arrows indicate relative increase or upregulation; downward arrows indicate relative decrease or downregulation.

This review addresses four linked questions. First, how does the host’s immune state influence the response to immunotherapy? Second, is psychological distress associated with measurable immune dysregulation in cancer? Third, which mechanisms may connect distress-related signaling to impaired antitumor immunity? Finally, how might this framework inform biomarker development and the design of host-modulation trials? We argue that psychological distress should be considered neither a vague psychosocial background factor nor a substitute for tumor biology, but a biologically relevant host-state variable that may help explain part of the variability in cancer immunotherapy response. This conceptual framework is summarized in **Figure 1**.

## 2 Host immune state as a determinant of immunotherapy response

Immune checkpoint inhibitors do not generate *de novo* antitumor immunity; rather, they release inhibitory constraints on pre-existing or inducible immune responses. Their efficacy, therefore, depends on whether the host retains immune cells capable of activation, proliferation, trafficking, and cytotoxic function once inhibitory signaling is relieved (1–5), thus helping to explain why tumor-intrinsic biomarkers alone are often insufficient.

Even when a tumor shows potentially favorable features, such as PD-L1 expression or neoantigen-related immunogenicity, a response can still fail if the systemic immune context is profoundly suppressed, ineffectively inflamed, or functionally exhausted (1–5). Importantly, PD-L1 expression and inflammatory activation are not inherently beneficial in cancer; rather, in some settings, they indicate a pre-existing but restrained immune response that is more amenable to therapeutic reinvigoration by checkpoint blockade. Conversely, some patients with imperfect tumor biomarker profiles still derive clinical benefit, suggesting that host immune competence contributes meaningfully to treatment responsiveness (1, 4, 5).

Peripheral blood studies support this host-side perspective. Expansion of peripheral PD-1^+^ CD8^+^ T cells after PD-1 blockade tracks with response in lung cancer patients (5). More recent work shows that baseline peripheral CD8^+^ T-cell activation potency, lymphocyte subsets, and circulating CD4^+^ T-cell profiles distinguish responders from non-responders in specific treatment settings (6–8). Multidimensional peripheral blood immunoprofiling further identifies distinct immunotypes with differential response characteristics, suggesting that host-state signals are unlikely to be reducible to a single cell type or marker (9).

Routine inflammatory indices point in the same direction. Meta-analytic data indicate that the neutrophil-to-lymphocyte ratio measured before treatment is associated with immunotherapy efficacy across advanced cancers (11). Cytokine meta-analyses likewise show that lower pretreatment IL-6 and IL-8, together with more favorable on-treatment inflammatory dynamics, are associated with better outcomes (12). Although these markers are nonspecific, their repeated association with outcomes supports the broader idea that immunotherapy depends on host biology that extends beyond the tumor itself.

Conceptual and translational reviews reinforce this view by situating cancer within an organism-wide immune landscape and emphasizing that peripheral blood biomarkers complement tissue markers, capturing the dynamic host context in which checkpoint therapy operates (1, 4, 13). Taken together, these studies support a broader framework for immunotherapy responsiveness: tumor biology remains essential, but therapeutic benefit also depends on whether the host immune system is sufficiently competent, coordinated, and recoverable to respond to treatment.

If host immune state influences immunotherapy efficacy, then factors that shift that state become clinically relevant. Psychological distress is one such candidate. The next section, therefore, examines whether distress-related states in cancer patients are associated with measurable endocrine, inflammatory, and immune changes that plausibly influence antitumor competence.

## 3 Psychological distress is linked to immune dysregulation in cancer

For psychological distress to be considered a plausible determinant of immunotherapy responsiveness, it must be linked to measurable host biology rather than only to subjective burden. In cancer patient populations, this requirement is increasingly supported by clinical evidence. Distress-related states—including depressive symptoms, anxiety, persistent psychological distress, and low social support—have been associated with altered endocrine regulation, inflammatory activation, impaired innate immune function, and broader immune imbalance (14, 19–28).

One of the earliest and most influential lines of evidence comes from studies of natural killer cell activity. In ovarian cancer, greater psychological distress and lower social support were associated with lower natural killer cell activity not only in peripheral blood mononuclear cells, but also in tumor-infiltrating and ascites-derived lymphocyte populations (14). This finding remains important because it linked psychosocial variables to immune changes in tumor-relevant compartments rather than only in peripheral blood.

Comparable observations have been reported in breast cancer-related settings. Greater psychological stress during diagnostic breast biopsy was associated with reduced natural killer cell activity and altered cytokine profiles, including lower interferon-γ (IFN-γ) production (19). In women newly diagnosed with early-stage breast cancer, mindfulness-based stress reduction was accompanied by partial recovery of selected immune measures over time (20). These intervention data do not establish oncologic benefit, but they support the view that at least some distress-linked immune changes may be biologically dynamic rather than fixed.

The endocrine-inflammatory axis has also been documented across multiple oncology cohorts. Depression in cancer patients has been associated with increased plasma IL-6 and altered hypothalamic-pituitary-adrenal axis function (21). Elevated IL-6, together with hypothalamic–pituitary–adrenal (HPA) axis activation, has likewise been observed in breast cancer patients with major depressive disorder (22). In metastatic renal cell carcinoma, depressive symptoms and flattened diurnal cortisol rhythms predicted worse survival, while leukocyte transcriptional analyses implicated pro-inflammatory and metastasis-related programs (23). This combination of psychological, endocrine, immune, and clinical readouts is especially informative, as it places host biology and outcomes within a single analytic framework.

More recent work has strengthened the inflammatory dimension of this relationship. Meta-analytic evidence links cancer-related depressive symptoms to elevated inflammatory markers across studies (26). In breast cancer survivors, psychological distress was associated with higher IL-1β, neutrophil-to-lymphocyte ratio, granulocyte-to-lymphocyte ratio, and systemic immune-inflammation index (27). Although this was not an immunotherapy study, it remains relevant because it linked distress to the same peripheral inflammatory markers that are increasingly studied as host-state biomarkers in oncology.

Taken together, these findings support a consistent interpretation: psychological distress in cancer is often associated with a host state marked by altered cortisol regulation, inflammatory activation, and reduced cellular immune competence. The exact pattern varies by tumor type, stage, treatment context, and the instrument used to capture distress. Still, the convergence of endocrine, inflammatory, and immune findings makes it difficult to treat distress as merely a parallel psychosocial burden without biological consequence (14, 19–28).

This notion has direct implications for immunotherapy. Checkpoint blockade depends on the presence of immune cells capable of activation and functional rescue within a host environment not dominated by ineffective inflammation or suppressive signaling. Distress-related states associated with elevated IL-6, altered cortisol rhythms, reduced natural killer cell function, and broader immune dysregulation may therefore tip the scales toward a less favorable host context for successful treatment. The evidence reviewed here does not establish that distress directly causes impaired immunotherapy efficacy. It does, however, introduce an essential intermediate step: in cancer patients, distress is repeatedly associated with biological changes relevant to antitumor immune competence.

## 4 Mechanisms linking distress to antitumor immunity

The clinical associations delineated above become more compelling when considered alongside mechanistic studies showing that stress-related signaling can directly impair antitumor immunity. These mechanisms do not imply that psychological distress overrides tumor biology. Rather, they support a model in which distress-related neuroendocrine and neural signals reshape the host context in which tumor-immune interactions occur. Glucocorticoid signaling, adrenergic signaling, and neural regulation of the tumor microenvironment converge on immune-cell fitness, antigen presentation, inhibitory signaling, and immune surveillance (29–33, 41–45).

### 4.1 Glucocorticoid signaling and immune dysfunction

In murine tumor models, endogenous glucocorticoid signaling contributes to the differentiation of dysfunctional CD8^+^ tumor-infiltrating lymphocytes in the tumor microenvironment (33). Glucocorticoid receptor activity increases along the gradient from more functional to more dysfunctional CD8^+^ T-cell states, transactivates IL-10 and multiple checkpoint receptors, and reduces the efficacy of immune checkpoint blockade. Tumor-associated monocyte–macrophage lineage cells also act as a local source of glucocorticoids, indicating that glucocorticoid-rich signaling can become embedded within the tumor immune niche.

In pancreatic cancer models, glucocorticoid receptor signaling increases PD-L1 while repressing MHC-I, thereby enhancing inhibitory signaling and reducing tumor visibility to cytotoxic T cells (29). Glucocorticoid receptor inhibition or depletion increases cytotoxic T-cell infiltration and improves sensitivity to checkpoint therapy. Together, these studies show that glucocorticoid signaling acts on both sides of the tumor–immune interaction by weakening immune effector function and increasing tumor immune escape.

An additional mechanistic link between psychological stress and impaired antitumor immunity is the stress–glucocorticoid–TSC22D3 axis (45). In murine models, social defeat stress or exogenous glucocorticoid exposure increases corticosterone levels, induces Tsc22d3 expression in dendritic cells, suppresses type I IFN responses and IFN-γ- positive T-cell activation, and abolishes therapeutic tumor control. These effects are reversed by glucocorticoid receptor antagonism or dendritic-cell-specific Tsc22d3 deletion. Correlations among plasma cortisol levels, leukocyte TSC22D3 transcript expression, and negative mood in patients with cancer further support a potential translational link between stress biology and impaired antitumor immune priming.

Although prescribed corticosteroid exposure is not equivalent to psychological distress, both states engage overlapping glucocorticoid pathways. For that reason, baseline corticosteroid studies provide indirect but clinically relevant support for the idea that glucocorticoid-rich states weaken the conditions required for effective immunotherapy. In advanced non-small-cell lung cancer, baseline corticosteroid use at the start of PD-1 or PD-L1 blockade is associated with poorer outcomes. However, this relationship is difficult to interpret because steroids are often prescribed to patients with greater symptom burden, more advanced disease, or other adverse clinical features (41, 42).

A similar concern applies in neurooncology, where dexamethasone is frequently required to control cerebral edema. In glioblastoma, concurrent dexamethasone exposure during anti–PD-1 immune checkpoint blockade is associated with poorer outcomes (46). In immune-based virotherapy settings, high-dose dexamethasone reduces immune activation and survival benefit in CAN-2409 models, whereas in the DNX-2401 plus pembrolizumab trial, baseline steroid use is constrained by eligibility criteria, and treatment-related steroid exposure shows outcome-associated trends despite inadequate statistical power (47, 48). Consistent with this broader immune dependence, the CAN-3110 trial links response and survival to T-cell activation and immune fitness (49).

Taken together, these data do not establish equivalence between prescribed corticosteroids and distress-related glucocorticoid signaling. They do, however, strengthen the broader argument that glucocorticoid-rich states compromise the immune conditions required for effective immunotherapy.

### 4.2 Adrenergic signaling, T-cell metabolism, and exhaustion

Adrenergic signaling provides a second major mechanistic link between psychological distress and antitumor immune dysfunction. In murine immune cell activation models, β-adrenergic signaling blocks the metabolic reprogramming required for effective CD8^+^ T-cell activation, including glucose uptake, glycolysis, and mitochondrial function (30). This finding links adrenergic stress directly to the bioenergetic programs required for effector T-cell differentiation.

Chronic adrenergic stress also contributes to metabolic dysfunction and an exhausted phenotype in tumor-infiltrating T cells (31). In murine models, chronic stress promotes T-cell states associated with ineffective antitumor immunity and poor responsiveness to checkpoint therapy. Because metabolic exhaustion is a major barrier to effective checkpoint reinvigoration, these findings are directly relevant to immunotherapy.

Direct support for the checkpoint relevance of adrenergic stress comes from murine models in which housing temperature-induced β-adrenergic stress suppresses an effector CD8+ T-cell phenotype and undermines the efficacy of anti-PD-1 and anti-CTLA-4 therapy (44). β-blockade reverses these effects, supporting the interpretation that adrenergic signaling limits checkpoint inhibitor activity not only by altering T-cell biology but also by directly reducing therapeutic efficacy *in vivo*. This experimental framework is especially informative because it links a stress-related adrenergic state to both immune dysfunction and impaired checkpoint responsiveness.

Additional support comes from chronic stress models showing accelerated tumor progression that is mitigated by β-blockade. In hepatocellular carcinoma, chronic restraint stress promotes tumor growth and immune escape from T-cell surveillance, reduces MHC-I expression, increases PD-L1 expression, and implicates β-adrenergic signaling in these changes (32). Together, these studies support a coherent model in which adrenergic stress impairs antitumor immunity by weakening immune-cell function and increasing tumor immune evasion.

A related hematologic cancer model supports the pathway’s broader relevance. In an orthotopic human pre-B acute lymphoblastic leukemia model, chronic restraint stress accelerates leukemia burden and dissemination through a β-adrenergic mechanism blocked by propranolol, with evidence suggesting indirect regulation via host cells or the bone marrow microenvironment rather than direct norepinephrine-driven proliferation of leukemia cells (50).

### 4.3 Neural regulation of tumor immunity and tumor-nerve-immune crosstalk

The mechanistic framework broadens further when extended beyond endocrine signaling to direct neural regulation of tumor immunity. Cancer is increasingly understood not only as a disease of malignant and immune cells, but also as one involving active crosstalk with the nervous system. Tumor–nerve crosstalk is not limited to stress pathways. Autonomic and neuromodulatory signaling can directly support tumor progression, as shown by sympathetic/neurotrophin feedforward signaling in pancreatic cancer, autonomic nerve development in prostate cancer, and cholinergic neuronal activity promoting diffuse midline glioma growth through muscarinic signaling (51–53). Tumors recruit, reshape, and exploit neural inputs, while neural signaling alters immune surveillance and inflammatory tone (43). This concept is especially well developed in glioblastoma biology, but is not limited to it. Malignant cells integrate into neural circuits through neuron–cancer cell synaptic communication and activity-dependent pathways, such as BDNF–TrkB signaling, that promote malignant synaptic plasticity and tumor progression. Recent spatial and circuit-level studies further suggest that glioblastoma regions with enhanced neuronal connectivity show regional immunosuppression, linking neural co-option to immune contexture rather than tumor growth alone (54–57).

Recent work in head and neck squamous cell carcinoma shows that tumors co-opt inter-organ neuroimmune circuits to evade immune surveillance. Cancer cells engage a neuroimmune circuit that suppresses immune activity, and targeting nociceptive neurons or the ATF4–SLIT2–CGRP axis restores immune function and improves responsiveness to checkpoint blockade (58). In this model, tumor cells under immune stress activate ATF4, a stress-responsive transcription factor, which induces SLIT2, a secreted neuronal guidance cue that stimulates nociceptive neurons. These neurons then release CGRP, a sensory neuropeptide that remodels tumor-draining lymph nodes and impairs dendritic cell and T cell activation, being one of the strongest current examples linking neural signaling, immune escape, and immunotherapy outcome.

This concept is especially relevant to central nervous system malignancies, although the mechanisms differ from those in peripheral sensory neuron pathways. In glioblastoma, tumor cells receive glutamatergic synaptic input from neurons, and this neuronal integration promotes brain tumor progression (57). More recent work shows that glioma-driven neuronal circuit remodeling induces regional immunosuppression, including altered macrophage states and reduced antitumor immune activity (55). Brain metastases also exploit the neural niche: breast-to-brain metastatic cells use synaptic proximity and NMDAR signaling to support colonization and growth (59). In parallel, reciprocal interactions between astrocytes and innate immune cells promote neuroinflammation and brain metastasis through lipocalin-2 signaling (60). Preclinical models also show that chronic stress can alter neural plasticity, neuroinflammatory pathways, and monoaminergic regulation, including REDD1-dependent synaptic loss, type I interferon- and complement-dependent neuroinflammation, and noradrenergic regulation of dopaminergic stress resilience (61–63). Together, these studies support a broader principle: primary and metastatic brain tumors do not merely grow within neural tissue, but co-opt neural, glial, and immune programs that shape therapeutic responsiveness.

A recent study on glioma further strengthens the brain–body component of this framework by demonstrating that chronic stress promotes glioma growth through macrophage-mediated brain–bone marrow crosstalk (64). Mechanistically, chronic stress promoted sympathetic nervous system-dependent differentiation of ADRB2+ bone marrow monocytes into CD45+CD11b+C5aR1+ stress-associated macrophages that infiltrated gliomas, displayed CD36-associated lipid accumulation, and impaired phagocytic function. Genetic or pharmacologic inhibition of the C5aR axis reduced stress-associated macrophage abundance and attenuated stress-induced glioma growth, while stress-associated macrophage abundance correlated with self-reported stress levels and prognosis in glioma patients (65).

Neural regulation studies also support the idea that brain-to-body signals influence tumor immunity at the systems level. In mouse models, activation of the brain’s reward system boosts immunity (66) and modulates antitumor immunity in tumor-bearing animals (67). Although preclinical, these studies provide proof of principle that central neural state shapes peripheral immune function in ways relevant to tumor control.

Imaging studies in humans provide an additional translational bridge between psychological stress, neural activity, hematopoiesis, inflammation, and cancer outcome. Resting amygdalar metabolic activity measured by fluorine-18 fluorodeoxyglucose positron emission tomography combined with computed tomography (FDG-PET/CT) has been proposed as an imaging marker of stress-associated neural activity. It has been linked to bone marrow activity and inflammatory signaling in humans (68). In clinical cancer research, higher amygdalar activity measured at head and neck cancer staging independently predicted poorer survival. It was associated with hematopoietic tissue activity and systemic inflammation, supporting the concept of an amygdala–bone marrow–inflammatory axis in cancer (69). While these imaging data do not prove that psychological distress causes impaired immunotherapy response, they nevertheless provide an explanatory, clinically measurable neuroimmune axis that allows bridging patient-reported distress, central stress biology, hematopoietic activation, and systemic inflammation.

Taken together, the mechanistic evidence supports a multi-layered model. Glucocorticoid signaling promotes T-cell dysfunction and immune escape. Adrenergic signaling impairs metabolic fitness, drives exhaustion, and reprogram myeloid development. This framework is consistent with broader evidence that sympathetic nervous system signaling can regulate multiple components of the tumor microenvironment and that stress-related neural and endocrine pathways influence cancer progression through immune, vascular, stromal, and metastatic mechanisms (70, 71). Neural and neuroimmune circuits shape local and systemic immune surveillance, including through sensory pathways linked to immunosuppressive mediators such as CGRP, tumor-associated neural remodeling, particularly in glioma, and stress-associated brain–bone marrow signaling. These pathways do not operate identically across tumors or patients. Still, they converge on the same general outcome: a host immune state that is less permissive for effective antitumor immunity and less favorable for successful checkpoint blockade (29–33, 43–45, 55, 57, 58, 67). Representative mechanistic studies linking neuroendocrine and neural signaling to antitumor immunity and immunotherapy response are summarized in **Table 1**.

**Table 1:** Mechanistic studies linking neuroendocrine and neural signaling to antitumor immunity and immunotherapy response.

Study	Model	Pathway/axis	Immune effect	Tumor/treatment effect	Relevance to immunotherapy
Acharya et al. (33)	Murine tumor models; tumor-infiltrating CD8+ T-cell analyses	Endogenous glucocorticoid receptor signaling	Promoted dysfunctional CD8+ T-cell differentiation and checkpoint/IL-10-associated programs	Reduced effective antitumor immune control	Shows that glucocorticoid signaling can drive T-cell states that are less amenable to checkpoint reinvigoration
Deng et al. (29)	Pancreatic cancer cell lines and murine tumor models	Glucocorticoid receptor signaling in tumor cells	Indirectly reduced cytotoxic T-cell infiltration by altering tumor immune phenotype.	Increased PD-L1, reduced MHC-I, promoted immune evasion, and immunotherapy resistance.	Demonstrates a tumor-cell-intrinsic mechanism by which glucocorticoid signaling may reduce checkpoint responsiveness
Qiao et al. (30)	Murine CD8+ T-cell activation studies	β-adrenergic signaling	Blocked metabolic reprogramming during activation; reduced glucose uptake, glycolysis, and mitochondrial function	Weakened effector differentiation potential	Links adrenergic stress directly to impaired metabolic fitness of antitumor T cells
Qiao et al. (31)	Murine tumor models; tumor-infiltrating T-cell analyses	Chronic adrenergic stress/β-adrenergic signaling	Promoted exhausted and metabolically dysfunctional T-cell states in the tumor microenvironment	Reduced effective antitumor immunity	Supports the idea that chronic stress can generate T-cell states less responsive to checkpoint rescue
Chen et al. (32)	Chronic stress hepatocellular carcinoma model	Stress-associated β-adrenergic signaling with MAPK/STAT1–IRF1 and eIF2α-linked immune-evasion pathways	Reduced T-cell surveillance	Suppressed MHC-I, increased PD-L1, promoted tumor growth and immune escape	Integrates chronic stress exposure with immune escape mechanisms relevant to checkpoint resistance
Yang et al. (45)	Murine stress models with therapy-induced antitumor immunity readouts	Stress–glucocorticoid–TSC22D3 axis	Impaired therapy-induced anticancer immunosurveillance	Abolished therapeutic tumor control under stress or glucocorticoid exposure	Strong evidence that stress-induced glucocorticoid signaling can directly undermine treatment-induced antitumor immunity
Bucsek et al. (44)	Murine tumor models under housing-related adrenergic stress	β2-adrenergic receptor signaling	Suppressed effector CD8+ T-cell phenotype and increased PD-1-high dysfunctional features	Undermined anti-PD-1/anti-CTLA-4 efficacy; β-blockade improved response	Provides direct preclinical evidence that adrenergic stress signaling can reduce checkpoint inhibitor efficacy
Ben-Shaanan et al. (66)	Mouse reward-circuit activation model	Brain reward system signaling	Enhanced innate and adaptive immune responses via sympathetic pathways	Not tumor-specific in this study	Provides proof of principle that the central neural state can modulate peripheral immune competence
Ben-Shaanan et al. (67)	Tumor-bearing mouse models	Brain reward system signaling	Enhanced antitumor immune responses	Reduced tumor burden/improved antitumor control	Demonstrates that brain-state manipulation can affect tumor immunity *in vivo*
Zhang et al. (58)	Cancer models with neuroimmune circuit analyses	Inter-organ neuroimmune circuit; ATF4–SLIT2–CGRP axis; nociceptive neuron signaling	Suppressed immune surveillance; immune function restored after pathway targeting	Promoted immune escape; targeting the circuit improved ICB responses	Strong evidence that tumors can co-opt a neuroimmune circuit and that disrupting it may enhance immunotherapy responsiveness
Nejo et al. (55)	Glioma models	Glioma–neuronal circuit remodeling	Regional immunosuppression; altered macrophage states; reduced antitumor immune activity	Supports glioma growth through neural circuit remodeling	Directly supports the idea that neural remodeling can create immune-suppressed tumor regions that may limit immunotherapy responsiveness
Venkataramani et al. (57)	Glioma models	Glutamatergic neuron–glioma synaptic input	Not primarily immune-focused	Neuronal synaptic input drives glioma progression	Shows that gliomas integrate into neural circuits, providing a biological basis for neural influence on therapeutic response
Zeng et al. (59)	Breast-to-brain metastasis models	Synaptic proximity–NMDAR signaling	Not primarily immune-focused	NMDAR signaling supports brain metastatic colonization and growth	Extends tumor–neural co-option to brain metastasis; relevant as a CNS niche mechanism that may shape treatment response
Adler et al. (60)	Brain metastasis models	Astrocyte–innate immune cell–lipocalin-2 axis	Innate immune–astrocyte crosstalk promotes neuroinflammation	Facilitates brain metastasis progression	Supports neural/glial/immune niche remodeling in metastasis, but is less directly linked to checkpoint response
Lamkin et al. (50)	Pre-B acute lymphoblastic leukemia model	Chronic restraint stress/β-adrenergic signaling	Indirect host-cell or bone marrow microenvironment-mediated effect	Increased leukemia burden and dissemination; blocked by propranolol	Extends stress-adrenergic host-state biology beyond solid tumors into hematologic malignancy models
Yang et al. (45)	Glioma mouse models and glioma patient samples	Chronic stress–sympathetic nervous system–ADRB2+ bone marrow monocyte–C5aR1+ stress-associated macrophage axis	Increased C5aR1+ stress-associated macrophages, decreased NK cells, CD36-associated lipid accumulation, and impaired macrophage phagocytic function	Accelerated glioma growth and shortened survival under chronic stress; macrophage-specific C5ar1 deletion or pharmacologic C5aR inhibition attenuated stress-induced glioma growth	Strong mechanistic evidence that chronic stress can reprogram bone marrow-derived myeloid cells and promote glioma progression through a targetable brain–bone marrow neuroimmune pathway

ADRB2, adrenergic receptor beta 2 ATF4, activating transcription factor 4; C5aR, complement component 5a receptor; C5aR1, complement component 5a receptor 1;CGRP, calcitonin gene-related peptide; CNS, central nervous system; CTLA-4, cytotoxic T-lymphocyte-associated protein 4;CCL3, C-C motif chemokine ligand 3**;** CD8+, cluster of differentiation 8-positive; CD36, cluster of differentiation 36; eIF2α, eukaryotic translation initiation factor 2 alpha; ICB, immune checkpoint blockade; IL-10, interleukin-10; IRF1, interferon regulatory factor 1; M1, classically activated macrophage-like phenotype; MAPK, mitogen-activated protein kinase; MHC-I, major histocompatibility complex class I; NMDAR, N-methyl-D-aspartate receptor; NK, natural killer; PD-1, programmed cell death protein 1; PD-L1, programmed death-ligand 1; SAM, stress-associated macrophage; SLIT2, slit guidance ligand 2; SNS, sympathetic nervous system; STAT1, signal transducer and activator of transcription 1; TSC22D3, TSC22 domain family member 3.

## 5 Clinical evidence linking emotional distress before immunotherapy initiation to immune checkpoint inhibitor outcomes

Direct clinical evidence linking psychological distress to immunotherapy outcome remains limited, but it has moved beyond speculation. The most informative studies are prospective or quasi-prospective cohorts in which distress is assessed before treatment initiation, patients are followed during immune checkpoint inhibitor therapy, and biological or survival outcomes are recorded. Across this literature, the most consistent pattern is that emotional distress assessed before checkpoint blockade initiation is associated with poorer response and survival during treatment (34–40). Representative clinical studies linking pretreatment emotional distress to outcomes during immune checkpoint inhibitor therapy are summarized in **Table 2**.

**Table 2:** Clinical studies linking pretreatment emotional distress to immunotherapy outcomes.

Study	Cancer type/setting	N	Study design	Treatment	Distress instrument and timing	Biological measures	Clinical endpoint(s)	Main finding	Confounding/adjustment considerations	Prognostic vs predictive interpretation
Zeng et al. (39)	Advanced non-small-cell lung cancer	227	Prospective observational cohort	First-line ICI-based therapy	PHQ-9 and GAD-7; pretreatment	Cortisol	ORR, PFS, OS	Pretreatment emotional distress was associated with lower ORR, shorter PFS, and worse OS.	Observational design; residual confounding by symptom burden, disease burden, sleep/fatigue, performance status, and corticosteroid exposure remains possible.	Supports prognostic/associative relevance during ICI therapy; predictive value for differential ICI benefit remains unproven.
Huang et al. (34)	Advanced inoperable gastroesophageal cancer	84	Prospective single-center observational cohort	First-line ICI-based therapy	PHQ-9 and GAD-7; pretreatment	Peripheral inflammatory and nutritional biomarkers	PFS, DCR	Pretreatment distress was associated with poorer PFS and lower disease control; adding peripheral biomarkers improved predictive performance.	Biomarker-integrated model, but single-center cohort; potential confounding by inflammatory/nutritional status, tumor burden, performance status, and treatment heterogeneity requires external validation.	Supports distress as part of a composite host-state risk profile; predictive value remains to be validated.
Tian et al. (35)	Advanced gastric cancer	104	Prospective cohort with multivariable analysis and propensity score matching	ICI-based combination therapy	PHQ-9 and GAD-7; pretreatment	ACTH and cortisol in a subset	DCR, PFS, OS	Distress was associated with higher risk of progression and death and lower disease control.	Multivariable and propensity-score approaches were used; relevant covariates included clinical, treatment, endocrine, inflammatory, and tumor-related factors. Endocrine biomarker data were limited/incomplete.	Strengthens prognostic association after adjustment, but does not establish ICI-specific predictive value.
Fraterman et al. (40)	Stage IIIB–D melanoma, neoadjuvant setting	88	*Post hoc* analysis of a phase 2 trial	Neoadjuvant combination ICB: ipilimumab plus nivolumab	EORTC QLQ-C30 emotional functioning scale; pretreatment	Exploratory inflammatory and T-cell activation markers; IFN-γ signature and tumor mutational burden considered	Major pathologic response, RFS, DMFS	Pretreatment distress was associated with lower major pathologic response and worse relapse-free and distant metastasis-free survival.	*Post hoc* analysis; emotional distress was inferred from an emotional functioning scale rather than a dedicated distress instrument; the perioperative trial context limits generalizability.	Suggests association with neoadjuvant ICB outcome, but lacks a non-ICB comparator needed to prove predictive relevance.
Wang et al. (36)	Recurrent high-grade glioma	75	Observational translational cohort in a specialized disease setting	Tislelizumab-based combination therapy: tislelizumab plus bevacizumab plus temozolomide	PHQ-9 and GAD-7; pretreatment	TISF-ctDNA, tumor immunohistochemistry, and inflammatory blood indices	ORR, PFS, OS	Distress was associated with impaired efficacy of checkpoint blockade-based therapy.	Glioma-specific confounding factors include neurologic symptoms, recurrence burden, corticosteroid exposure, edema control, prior therapy, and combined bevacizumab/temozolomide treatment.	Hypothesis-generating association in an immunologically specialized setting; predictive value remains unproven.
Cohen et al. (37)	Mixed advanced cancers	62	Small exploratory observational cohort	ICI therapy, mixed regimens	Questionnaire-based perceived health, HRQoL, distress, and sleep measures; baseline around treatment initiation	IL-6, TNF-α, soluble CTLA-4, soluble PD-1	Treatment response	Better perceived health was associated with better response; distress-related variables were associated with less favorable biomarker profiles.	Small and heterogeneous cohort; not powered to establish distress as an independent predictor; limited ability to adjust systematically for disease burden, treatment type, performance status, or prior therapy.	Exploratory biomarker association; useful for hypothesis generation, not for prognostic or predictive validation.
Li et al. (38)	Advanced NSCLC, pooled trial cohorts	4, 632	Individual patient data meta-analysis of trial cohorts	ICIs or chemotherapy across pooled trial cohorts	EORTC QLQ-C30 emotional functioning scale; baseline/pretreatment	Not primary	PFS, OS	Emotional distress was associated with worse survival outcomes, but appeared prognostic rather than specifically predictive for ICIs.	Strengthened by individual patient data and trial-derived cohorts, but limited to NSCLC and dependent on trial-level harmonization and available QLQ-C30 data.	Most directly addresses prognostic vs predictive distinction; supports prognostic relevance more strongly than ICI-specific predictive value.
Derry-Vick H (73)	Mixed advanced cancers; anti-PD-1/PD-L1-treated cohort	913	Multicenter retrospective real-world cohort	Anti-PD-1 or anti-PD-L1 monotherapy	Pre-existing anxiety or depression diagnosis at ICI initiation, abstracted from medical charts	Not primary	irAEs, OS, TTF	Anxiety diagnosis was associated with a higher likelihood of irAEs and better OS; depression diagnosis was not significantly associated with irAEs, OS, or TTF.	Adjusted for sociodemographic and clinical factors; chart-based diagnoses may not reflect standardized pretreatment distress, and residual confounding remains possible.	Large diagnostic cohort showing heterogeneous associations; supports caution rather than predictive interpretation.

ACTH, adrenocorticotropic hormone; CTLA-4, cytotoxic T-lymphocyte-associated protein 4; DCR, disease control rate; DMFS, distant metastasis-free survival; EORTC, European Organisation for Research and Treatment of Cancer; GAD-7, Generalized Anxiety Disorder 7-item scale; HRQoL, health-related quality of life; ICB, immune checkpoint blockade; ICI, immune checkpoint inhibitor; IL-6, interleukin-6; IPD, individual patient data; irAEs, immune-related adverse events; NSCLC, non-small-cell lung cancer; ORR, objective response rate; OS, overall survival; PD-1, programmed cell death protein 1; PD-L1, programmed death-ligand 1; PFS, progression-free survival; PHQ-9, Patient Health Questionnaire-9; QLQ-C30, Quality of Life Questionnaire Core 30; RFS, relapse-free survival; TISF-ctDNA, tumor in situ fluid circulating tumor DNA; TNF-α, tumor necrosis factor alpha; TTF, time to treatment failure.

### 5.1 Prognostic versus predictive interpretation and confounding

A central limitation of the current clinical literature is that most available studies support a prognostic rather than a definitive predictive interpretation of psychological distress. A prognostic marker is associated with outcome irrespective of treatment type, whereas a predictive marker identifies differential benefit from a specific therapy. Demonstrating predictive relevance, therefore, requires comparison across treatment contexts, ideally within randomized datasets, and formal testing for an interaction between distress status and treatment effect (72). By this standard, pretreatment of emotional distress cannot yet be considered a validated predictive biomarker of immune checkpoint inhibitor therapy benefit. The current data more strongly support distress as a host-state correlate associated with poorer outcomes during immunotherapy. At the same time, the extent to which it specifically modifies the benefit of checkpoint blockade remains unresolved (34–40). A recent multicenter cohort broadens this evidence base but reinforces the need for caution. In 913 patients treated with anti-PD-1 or anti-PD-L1 monotherapy, chart-documented anxiety and depression showed heterogeneous associations with outcomes: anxiety was associated with higher immune-related adverse events and longer overall survival, whereas depression showed no significant association with immune-related adverse events, overall survival, or time to treatment failure (73). As this study used clinical diagnoses rather than standardized patient-reported pretreatment distress instruments, it should be interpreted as important real-world evidence of heterogeneity, not as direct confirmation of distress as an ICI predictive biomarker. This distinction is important because emotional distress in patients with advanced cancer is strongly intertwined with clinical and biological factors that also influence immunotherapy outcomes. Pain, fatigue, sleep disruption, reduced performance status, corticosteroid exposure, tumor burden, prior treatment intensity, nutritional decline, and socioeconomic adversity may all increase distress and independently worsen cancer outcomes. These variables may act as confounders, mediators, or effect modifiers. For example, corticosteroid exposure can reflect more aggressive disease or symptom burden while also engaging glucocorticoid pathways that may suppress antitumor immunity (41, 42, 46–48). Similarly, poor performance status or high inflammatory burden may both increase emotional distress and reduce immune competence, making causal direction difficult to assign.

The studies summarized in **Table 2** vary substantially in how they address these problems. Some cohorts include multivariable adjustment or propensity score approaches, whereas others are exploratory and underpowered for independent predictive analysis (34–40). Future studies should therefore pre-specify a core set of confounders, as listed above. Ideally, studies should also incorporate serial psychological and biological measurements, allowing investigators to distinguish baseline vulnerability from treatment-emergent decline. At present, the most defensible conclusion is that pretreatment emotional distress identifies a clinically relevant and biologically plausible high-risk host state, but does not yet establish that distress causally determines checkpoint resistance or predicts differential benefit from immune checkpoint blockade.

An important limitation of the current clinical evidence is its uneven coverage of cancer types. At present, the strongest distress–immunotherapy outcome data come from advanced non-small-cell lung cancer, gastroesophageal and gastric cancers, melanoma, recurrent high-grade glioma, and small mixed-cancer cohorts (34–40). This distribution does not mirror the broader psycho-oncology literature. For example, breast and ovarian cancers have contributed substantially to evidence linking psychological distress, social isolation, cortisol dysregulation, inflammation, and impaired cellular immunity in cancer patients (14, 19–28), yet corresponding studies directly testing pretreatment distress against immune checkpoint inhibitor outcomes remain limited or absent. Similarly, colorectal cancer, renal cell carcinoma, and head and neck cancer are clinically prevalent immunotherapy populations. Still, they are underrepresented in studies that combine standardized pretreatment distress assessment with immune checkpoint inhibitor response, progression-free survival, or overall survival. Colorectal cancer is a particularly important gap as stress interacts with gut barrier and mucosal immune pathways. Experimental evidence shows, e.g., that psychological stress can impair IL-22-driven protective gut mucosal immunity against colonizing pathobionts, suggesting a plausible interface between stress biology, the microbiome–mucosal immune axis, and immunotherapy-relevant host state (74).

Head and neck cancer illustrates the distinction between emerging psycho-oncology data in immunotherapy-treated populations and direct pretreatment distress–efficacy evidence. A recent cross-sectional study of 232 advanced head and neck cancer patients used the Hospital Anxiety and Depression Scale and propensity score matching to compare posttreatment emotional status among patients receiving chemoradiotherapy with or without immune checkpoint blockade (75). Although this study is relevant as it shows that anxiety and depression are being studied in immune checkpoint blockade-treated head and neck cancer populations, it assessed emotional status after treatment. It did not test whether pretreatment distress predicted checkpoint response, progression-free survival, or overall survival. It should therefore be viewed as emerging supportive context rather than as direct evidence for pretreatment distress as a prognostic or predictive immunotherapy biomarker.

The absence of several tumor types from **Table 2** should not be interpreted as evidence that distress-linked host biology is irrelevant in these cancers, but rather as evidence that the necessary prospective immunotherapy cohorts have not yet been developed. Population-level evidence further suggests that psychological distress is associated with site-specific cancer mortality. However, such data do not address immunotherapy-specific response. They should therefore be interpreted in the epidemiologic context rather than as evidence of ICI prediction (76).

Future studies should prioritize tumor types in which immune-based therapies are already clinically established or expanding, but in which pretreatment distress–immunotherapy outcome data remain limited or absent. These include triple-negative breast cancer treated with immune checkpoint blockade, mismatch repair-deficient or microsatellite instability-high colorectal cancer, renal cell carcinoma, head and neck squamous cell carcinoma, and hematologic malignancies treated with cellular therapies such as CAR T cells (50, 77). These cohorts should use harmonized psychological instruments, capture core confounders, and include endocrine, inflammatory, and immune readouts so that distress–immune biology can be compared across tumor contexts rather than inferred from isolated cohorts.

After this methodological framing, the strongest current clinical anchor comes from advanced non-small-cell lung cancer. Emotional distress assessed before first-line immune checkpoint inhibitor treatment is associated with shorter progression-free survival, lower objective response rate, and worse overall survival. Higher distress is also associated with higher circulating cortisol, providing a biologically relevant endocrine correlate (39).

A similar pattern appears in upper gastrointestinal cancers. In advanced inoperable gastroesophageal cancer, emotional distress present before checkpoint inhibitor initiation predicts poorer outcomes, and predictive performance improves when distress is considered together with peripheral biomarkers (34). In advanced gastric cancer, distress assessed before ICI-based combination therapy is associated with a higher risk of progression and death and with lower disease control, with the association remaining robust in multivariable analyses and propensity score–matched comparisons (35).

Additional evidence comes from melanoma in the neoadjuvant setting (40). In a *post hoc* analysis of the phase 2 PRADO trial in stage IIIB–D melanoma, emotional distress assessed before neoadjuvant ipilimumab plus nivolumab, using the EORTC QLQ-C30 emotional functioning scale, is associated with a lower likelihood of achieving major pathologic response and with worse 2-year relapse-free and distant metastasis-free survival, even after adjustment for IFN-γ signature and tumor mutational burden. Although this analysis is *post hoc* and conducted in a perioperative rather than advanced metastatic setting, it extends the distress–immunotherapy association to melanoma. It suggests that the signal is not confined to lung or upper gastrointestinal cancers.

The pattern also extends to recurrent high-grade glioma. In one recent study, emotional distress assessed before checkpoint blockade-based treatment was associated with impaired treatment efficacy. It was analyzed alongside *in situ* tumor fluid, circulating tumor DNA, and tumor-associated measures (36). Although still early-stage, this finding suggests that the distress–outcome relationship extends even to cancers with highly specialized, suppressive immune microenvironments.

A smaller mixed-cancer exploratory cohort provides a useful complement to these disease-specific studies (37). Psychological measures are analyzed alongside circulating immune-related factors, including IL-6 and soluble checkpoint-related molecules. Better perceived health status is associated with better treatment response, whereas distress and sleep-related variables are associated with less favorable biomarker profiles. Because the cohort is small and heterogeneous, these findings remain hypothesis-generating.

Pooled evidence is also emerging. An individual patient data meta-analysis involving 4, 632 patients from advanced non-small-cell lung cancer trial cohorts shows that emotional distress is associated with worse survival outcomes. However, the signal appears to be prognostic rather than specifically predictive of immune checkpoint inhibition (38).

Taken together, current evidence supports the view that emotional distress assessed before immunotherapy initiation is a clinically relevant host-state correlate of outcomes during checkpoint therapy. However, as outlined above, the field remains limited by observational designs, heterogeneous measures of distress, confounding factors, and incomplete coverage of cancer types. What remains unresolved is whether distress specifically predicts differential benefit from immune checkpoint blockade or, more broadly, reflects an adverse host state and cancer course. Even so, when interpreted alongside the mechanistic literature, these patient studies make it increasingly difficult to treat distress as merely a concurrent quality-of-life variable unrelated to cancer immunotherapy (34–40).

## 6 A psycho-neuro-immune model of immunotherapy responsiveness

The evidence reviewed above supports a psycho-neuro-immune interpretation of cancer immunotherapy response that extends beyond tumor-centered biomarker models alone. Checkpoint efficacy depends on the host immune system remaining sufficiently competent and recoverable once inhibitory constraints are relieved (1–14). Psychological distress is associated with endocrine disruption, inflammatory activation, reduced natural killer cell function, and broader immune dysregulation (14, 19–28), while preclinical studies link stress-related signaling to impaired antitumor immunity (29–33, 44, 45, 50, 58, 66, 67). Together, these data support a model in which psychological distress identifies or contributes to a host state that is less permissive for effective immunotherapy.

A useful way to conceptualize this model is as a sequence. Patients entering treatment with higher emotional distress often show altered cortisol regulation, sympathetic arousal, or related neuroendocrine disturbance (22–24). These changes are associated with elevated inflammatory mediators, shifts in innate and adaptive immune activity, and reduced immune competence (14, 19–28). In parallel, preclinical models show that stress-linked signaling promotes dysfunctional CD8+ T-cell states, metabolic exhaustion, impaired antigen presentation, and increased expression of inhibitory ligands (29–33, 44, 45). In the context of checkpoint blockade, distress is therefore linked to a host condition in which the cellular substrate required for therapeutic reinvigoration is less available or less functional.

This framework also explains why inflammatory activation is not equivalent to effective antitumor immunity. A distressed patient may exhibit elevated cytokine levels or inflammatory indices yet remain poorly equipped to mount a successful checkpoint response. The relevant host state is not simply one of greater immune activity, but one in which immune activity is coordinated, metabolically fit, and capable of productive tumor-directed function (1, 4, 5). Chronic stress biology, in contrast, favors ineffective inflammation, checkpoint-associated dysfunction, myeloid skewing, reduced natural killer cell function, and exhausted or metabolically compromised T cells (14, 27, 29–33, 44, 45).

The neural dimension further broadens this interpretation. Emerging data indicate that the relationship between the brain and the tumor is not unidirectional (43, 58). Psychological and central nervous system states influence peripheral immune regulation, while experimental studies show that some tumors recruit and exploit neural and inter-organ neuroimmune circuits that suppress immune surveillance (58). These layers are not yet fully mapped in patients, but the available evidence supports a multi-system framework.

This perspective also helps interpret the current clinical literature. Observational cohorts do not prove that distress causes checkpoint resistance (34–40), but the mechanistic evidence makes the association biologically credible and harder to dismiss as incidental (29–33, 43–45, 50, 58, 66, 67). Thus, the next step is to test this framework prospectively through integrated biomarker strategies and host-modulation trial designs.

## 7 Translational implications for biomarkers and host-modulation trials

The value of a psycho-neuro-immune framework lies in its translational use: identifying measurable host states, improving risk stratification, and informing trial designs that test whether those states are modifiable in ways relevant to treatment outcome.

### 7.1 From distress screening to host-state stratification: a tiered biomarker framework

At present, the strongest translational implication is not that psychological distress should guide immunotherapy decisions on its own, but that it should be incorporated into prospective biomarker strategies as a candidate host-state variable alongside endocrine, inflammatory, immune, and, where available, imaging-based measures (4, 5, 12–14, 34–40). Glioma represents a particularly complex setting as depressive symptoms may reflect psychological distress, tumor-related neurobiology, neurologic impairment due to tumor burden, corticosteroid exposure, seizures, or treatment effects. Depression is frequent in adults with glioma and has been associated with clinical course and survival. However, establishing causal direction remains difficult (78–81). The immediate goal should therefore be operational rather than therapeutic: to define reproducible distress-linked host-state profiles before testing whether they are clinically modifiable.

A practical first step is to treat psychological assessment as part of host-state characterization before immunotherapy initiation rather than as a supportive-care add-on. Current immunotherapy cohorts indicate that emotional distress assessed before treatment is clinically informative, particularly when interpreted together with biological variables (34–40). If distress is to be studied as a biomarker-relevant host variable, it must be assessed reproducibly at defined time points and linked to specific biological readouts. A minimum psychological assessment should include a validated depression and anxiety instrument, such as the PHQ-9 and GAD-7, or an emotional functioning scale when embedded in oncology quality-of-life assessment (82–84). It should also record pain, fatigue, sleep disturbance, corticosteroid exposure, and performance status, immunotherapy treatment timing and regimen, as these factors may confound or mediate the association between distress and outcome (34–42).

The first tier should consist of feasible, interpretable, and scalable measures: standardized distress assessment; core clinical confounders; complete blood count-derived inflammatory indices such as the neutrophil-to-lymphocyte ratio and the systemic immune-inflammation index; C-reactive protein; and standardized cortisol measurement (11, 12, 22, 23, 27, 34–40). Cortisol is attractive because it is biologically interpretable and linked to mechanisms implicated in T-cell dysfunction and immune escape (23, 24, 29, 33, 41, 42, 45). Its utility, however, depends on rigorous measurement, including standardized sampling windows or repeated measurements. The inflammatory layer is currently the most advanced and scalable component of a psycho-neuro-immune biomarker strategy. However, its utility depends on rigorous measurement, including standardized sampling windows and, where feasible, repeated measurements.

Second tier, inflammatory markers are currently the most scalable component of this framework; distress and depression in cancer are repeatedly associated with elevated inflammatory mediators such as IL-6 and related indices (22, 23, 27, 28) and, in immunotherapy specifically, lower IL-6 and IL-8 levels before treatment initiation, together with more favorable inflammatory dynamics during treatment, are associated with better checkpoint outcomes (12).

A third biomarker tier should include peripheral immune phenotyping where resources permit. Circulating CD8+ T-cell activation, lymphocyte subset distribution, myeloid-cell features, natural killer cell frequency or function, and broader multidimensional immune profiles provide more direct information about immune competence than inflammatory indices alone (6–11). These readouts allow for the direct evaluation of distress-linked endocrine and inflammatory changes against immune-cell states more closely relevant to checkpoint reinvigoration. This tier is less universally scalable than blood count-derived markers, but it is important for mechanistic trials and biomarker-enriched studies.

Finally, the advanced translational tier should include imaging-derived neuroimmune biomarkers in selected cohorts. Resting amygdalar metabolic activity measured using fluorine-18 fluorodeoxyglucose positron emission tomography combined with computed tomography (FDG-PET/CT) has been proposed as an imaging marker of stress-associated neural activity. It has been linked to bone marrow activity and inflammatory signaling in humans (68). In oncology, higher amygdalar activity, as measured during head and neck cancer staging, independently predicted poorer survival. It was associated with hematopoietic tissue activity and systemic inflammation, supporting the concept of an amygdala–bone marrow–inflammatory axis in cancer (69). PET-based measures are not yet ready for routine distress-guided immunotherapy decisions. Still, they may be valuable in translational studies, as they provide an objective bridge among patient-reported distress, central stress biology, hematopoietic activation, and systemic inflammation.

**Figure 2:**
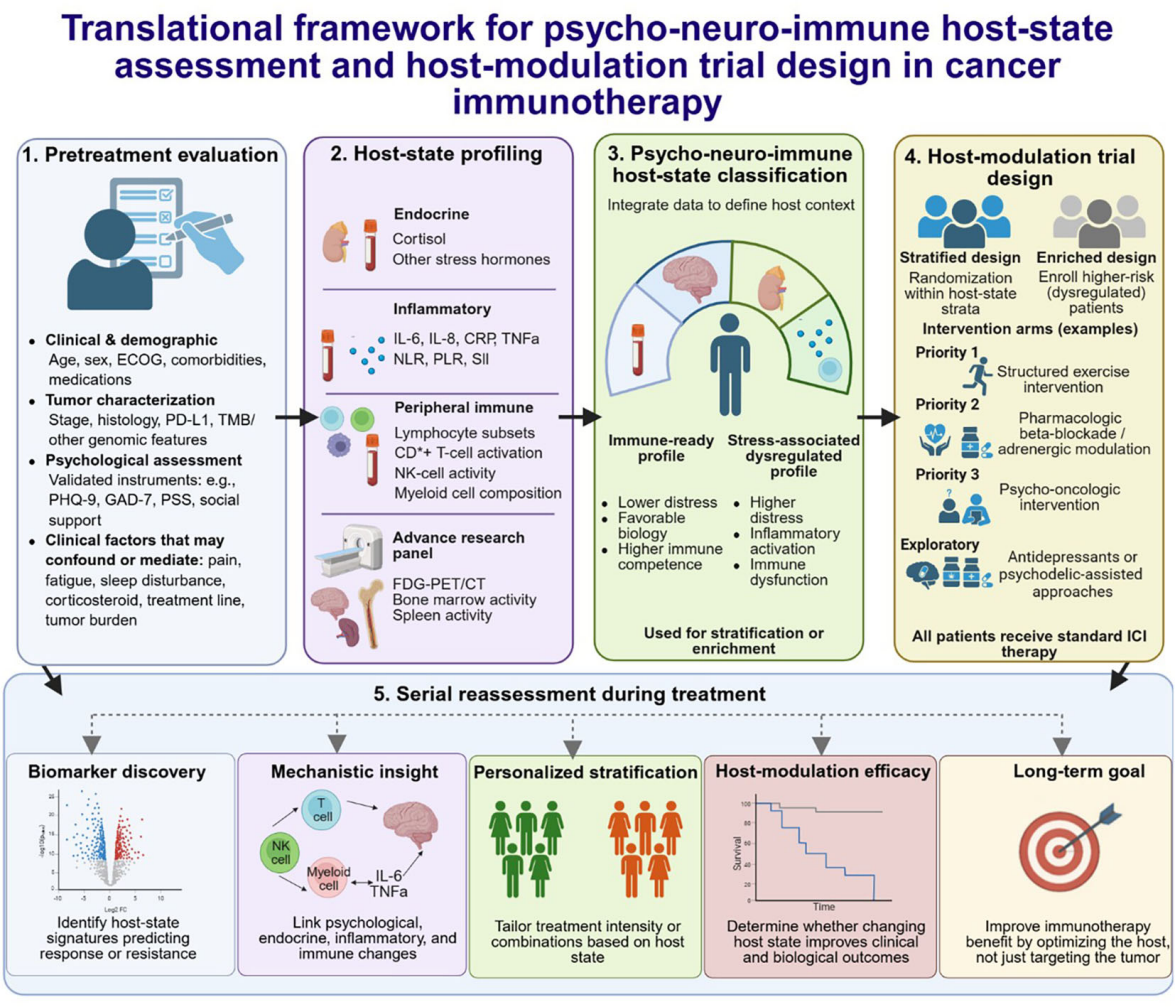
Translational framework for psycho-neuro-immune host-state assessment and host-modulation trial design in cancer immunotherapy. This workflow illustrates how psychological assessment could be integrated with clinical endocrine, inflammatory, immune, and imaging-based host-state profiling before initiating immune checkpoint inhibitor treatment. Evaluation before treatment would include a standardized distress assessment and conventional clinical and tumor characterization (box 1). Host-state profiling would include a minimum feasible panel of cortisol, C-reactive protein, complete blood count-derived inflammatory indices, and selected inflammatory mediators; mechanistic extensions may include cytokine profiling and immune-cell phenotyping, while advanced research panels may add FDG-PET/CT-derived activity in the amygdala, bone marrow, and spleen (box 2). These data could support classification into lower-risk immune-permissive and higher-risk distress-linked immune-restrictive host-state profiles (box 3). Such profiles could then be used for stratification or enrichment in prospective trials testing prioritized host-modulation strategies, including structured exercise, psycho-oncologic intervention, and biologically justified pharmacologic modulation such as adrenergic blockade (box 4). Serial reassessment during treatment would determine whether changes in host-state markers are associated with biological endpoints and clinical outcomes, including response, progression-free survival, treatment discontinuation, toxicity, quality of life, and overall survival (box 5). The framework is intended for hypothesis-driven prospective trial design and should not be used to select, withhold, or modify immunotherapy based solely on psychological distress. ICI, immune checkpoint inhibitor; β-AR, beta-adrenergic receptor; IL-6, interleukin-6; CRP, C-reactive protein; ECOG, Eastern Cooperative Oncology Group performance status; FDG-PET/CT, fluorine-18 fluorodeoxyglucose positron emission tomography combined with computed tomography; GAD-7, Generalized Anxiety Disorder 7-item scale; IL-8, interleukin-8; NLR, neutrophil-to-lymphocyte ratio; ORR, objective response rate; OS, overall survival; PFS, progression-free survival; PHQ-9, Patient Health Questionnaire-9; PLR, platelet-to-lymphocyte ratio; PSS, Perceived Stress Scale; SII, systemic immune-inflammation index; TMB, tumor mutational burden; TNF-α, tumor necrosis factor alpha.

This tiered approach does not assume that any single layer is decisive. Rather, it treats psychological state as one entry point into a broader host-state profile. A proposed translational workflow for integrating these measures into the design of immunotherapy biomarker and host-modulation trials is shown in **Figure 2**.

For near-term implementation, these biomarker layers should be prioritized based on feasibility, biological specificity, and current validation status. The minimum clinical–biological panel is the most scalable and should be implemented broadly, whereas peripheral immune phenotyping should be added in mechanistic phase II studies to improve biological resolution. Imaging-derived neuroimmune biomarkers, including FDG-PET/CT-derived amygdala, bone marrow, and spleen activity, provide a compelling bridge between central stress biology and systemic immune activation, but remain insufficiently validated for immunotherapy-specific prediction and should therefore be reserved for exploratory translational cohorts, particularly when such imaging is already clinically available. These considerations have direct implications for trial design. Current evidence does not justify selecting or withholding checkpoint therapy based on distress alone. It does justify embedding psycho-neuro-immune assessment into prospective immunotherapy trials. At a minimum, future studies should obtain psychological measures before treatment initiation, together with serial blood samples and standard clinical endpoints. More ambitious designs could then test whether patients with high-risk host-state profiles defined by distress plus endocrine, inflammatory, and immune abnormalities represent a subgroup with distinct treatment vulnerability (34–40, 85). Such studies should also prespecify stratification or adjustment for sex, age, tumor type, socioeconomic adversity, performance status, corticosteroid exposure, and disease burden, because these variables may moderate or confound the distress–immunity relationship.

Among currently plausible host-modulation strategies, structured exercise should be considered the highest-priority near-term intervention. It is feasible, scalable, biologically credible, and already supported by oncology outcome data (86–89). A pragmatic phase II design could enroll patients initiating immune checkpoint blockade, perform baseline distress and host-state profiling, and randomize patients with an unfavorable host-state profile to standard care versus standard care plus a structured exercise intervention. Co-primary endpoints should include both biological changes, such as improvements in inflammatory indices, cortisol rhythm, or immune-cell fitness, and clinical outcomes, such as objective response, progression-free survival, or treatment discontinuation. This design would test whether host-state modulation changes the biological context in which immunotherapy is delivered, rather than assuming that symptom improvement alone is sufficient.

A second priority approach is adrenergic modulation, particularly in biomarker-enriched settings. β-blockers are mechanistically appealing because adrenergic signaling is one of the clearest pathways linking stress biology to impaired antitumor immunity (30–32, 44). Emerging clinical data on β-blockers and immune checkpoint inhibitor outcomes remain exploratory (90, 91), so adrenergic modulation should not yet be treated as standard practice. However, a biomarker-driven trial could test whether patients with high sympathetic or inflammatory host-state signatures derive biological benefit from β-adrenergic blockade when combined with immunotherapy. Such a study would require careful safety monitoring, cardiovascular eligibility criteria, and predefined immune and clinical endpoints.

Psychotherapy and structured psycho-oncologic interventions remain important but should be framed differently. Their strongest current rationale is not that they are proven immunotherapy enhancers, but that they directly target psychological distress and have evidence of biological effects beyond symptom relief (20, 24, 25, 92–95). They are therefore reasonable candidates for trials in which the primary biological question is whether reducing distress can normalize endocrine, inflammatory, or immune parameters relevant to immunotherapy. Antidepressant repurposing also deserves exploratory consideration within host-modulation frameworks. Fluoxetine shows preclinical antitumor activity in glioblastoma (96), is associated with improved overall survival in observational cohorts of PD-1/PD-L1-treated cancer patients (97), and is currently being tested for effects on tumor immune cells in colorectal cancer (NCT06225011). Causality and immunotherapy-specific mechanisms, however, remain unresolved.

Other approaches, including psychedelic-assisted strategies, remain of interest, but the evidence is still too premature and too weakly tied to immunotherapy-specific endpoints to justify near-term translational emphasis (98, 99).

Caution remains essential. The field is not mature enough for aggressive therapeutic claims. Distress measures are heterogeneous, biomarker standardization is incomplete, and many candidate markers are nonspecific (11–13, 85). Interventions may improve symptoms without meaningfully altering immune competence, and the association between distress and outcome may still be partly confounded by disease burden, frailty, corticosteroid exposure, or treatment complexity (34–42, 46–48). The current agenda should therefore focus on integration, stratification, and biologically informed testing rather than implementation.

In summary, the translational value of this framework lies in shifting the field from a binary question, does distress matter?, to a more actionable one: which distress-linked host states are biologically relevant to immunotherapy, how should they be measured, and can they be modified in ways that improve treatment context or outcome? A tiered biomarker panel and prioritized host-modulation trials may move immunotherapy toward a more complete form of precision, one that accounts for both the tumor being treated and the host receiving treatment.

### 7.2 Moderators of distress–immune relationships: sex, age, tumor type, and social context

The biological consequences of psychological distress are unlikely to be uniform across patients. Sex, age, tumor type, comorbidities, and social determinants of health may all moderate the relationships among distress, neuroendocrine signaling, inflammation, and immune competence, and thus be of particular importance, as the distress–immune and distress–immunotherapy literatures are drawn from populations with different sex distributions and clinical contexts. Foundational psycho-oncology studies linking distress to immune dysregulation often include breast and ovarian cancer cohorts, whereas current immunotherapy outcome data are concentrated in lung, gastroesophageal/gastric, melanoma, glioma, and mixed-cancer cohorts (14, 19–28, 34–40). These populations differ not only in tumor biology, but also in sex distribution, symptom burden, treatment exposure, and likelihood of corticosteroid use.

Sex is a biologically plausible moderator because stress biology and immune function show that males and females can differ in hypothalamic–pituitary–adrenal axis reactivity, stress-induced inflammatory responses, and vulnerability to inflammation-associated depressive symptoms (100, 101). These differences are relevant to cancer immunotherapy because sex-based variation in innate and adaptive immunity may influence both antitumor immune responses and immune-related adverse events. Therefore, future distress–immunotherapy studies should not only adjust for sex, but also test whether distress-linked endocrine or inflammatory profiles differ by sex where sample size permits.

Age is another important moderator. Aging is associated with immuno-senescence, chronic low-grade inflammation, altered neuroendocrine regulation, and reduced immune reserve. Stress and aging may interact, because age-related changes in hypothalamic–pituitary–adrenal axis regulation and inflammatory control can make older adults more vulnerable to stress-associated immune dysregulation (102, 103). In immunotherapy cohorts, age may therefore influence both the baseline immune state and the biological consequences of distress. Future studies should examine whether distress-associated cortisol, inflammatory indices, lymphocyte composition, and myeloid features have different prognostic value in younger versus older patients.

Social determinants of health should also be treated as biological and methodological variables rather than as background descriptors. Socioeconomic adversity, financial toxicity, social isolation, limited access to supportive care, and treatment-related practical barriers can increase psychological distress. It may also shape inflammation, comorbidity, nutrition, physical activity, treatment adherence, and timing of care. In cancer populations, social determinants such as income, race, and access-related factors are associated with symptom burden during treatment, while broader survivorship literature documents disparities in cancer survival by socioeconomic status, education, poverty, and access to health insurance (104, 105). These factors may confound distress–outcome associations or identify patients whose distress reflects chronic structural adversity rather than only disease-related emotional burden.

Accordingly, future psycho-neuro-immune immunotherapy studies should pre-specify the capture of moderators and confounders. At minimum, models should adjust for sex, age, tumor type, performance status, corticosteroid exposure, disease burden, prior treatment line, pain, fatigue, sleep disturbance, and socioeconomic variables. When adequately powered, studies should stratify by sex and age and test whether socioeconomic adversity modifies the relationships among distress, inflammatory markers, immune phenotypes, and immunotherapy outcomes. Without this level of stratification, the field risks treating psychological distress as a uniform exposure when it may represent biologically and socially distinct host-state phenotypes.

## 8 Conclusion

Cancer immunotherapy has made clear that durable tumor control depends not only on tumor immunogenicity, but also on the condition of the host immune system. The evidence reviewed here supports the view that factors beyond tumor burden or baseline inflammation shape this host condition. Psychological distress is linked to endocrine, inflammatory, neural, and immune alterations relevant to antitumor competence (14, 19–33, 43–45, 58, 66, 67), while emerging clinical studies show that emotional distress assessed before checkpoint inhibitor treatment is associated with poorer outcomes across several cancer settings treated with immune checkpoint inhibitors (34–40). Together, these findings support a psycho-neuro-immune interpretation of immunotherapy responsiveness in which psychological distress identifies or contributes to a host state that is less favorable for therapeutic benefit.

The available evidence does not justify a causal claim that distress determines checkpoint resistance. It does, however, justify treating distress as biologically relevant to immunotherapy models. The convergence of clinical and mechanistic evidence now makes it difficult to regard distress as merely a parallel quality-of-life variable without implications for host immune readiness.

The central implication of this review is both conceptual and practical. The host state relevant to immunotherapy is multi-systemic rather than narrowly immune-defined. Tumor biology remains essential, but treatment efficacy also depends on psycho-neuro-endocrine-immune conditions that shape whether antitumor immunity can be effectively mobilized. Within this framework, psychological distress is best understood as a measurable component of host-state regulation that intersects with immunotherapy response.

This interpretation has direct consequences for research design. Future studies should move beyond asking whether distressed patients fare worse and instead define which distress-linked biological states are most relevant to treatment, how they are measured reproducibly, and whether they are modifiable (4, 5, 12, 13, 85). Prospective trials should integrate standardized psychological assessment with endocrine, inflammatory, and peripheral immune profiling before and during therapy. Biomarker-enriched host-modulation studies should then test whether altering the host context changes the biological and clinical conditions under which immunotherapy is delivered (86–95, 106).

A more complete model of immunotherapy response, therefore, needs to account not only for the tumor being treated, but also for the patient’s biological and psychological state. Optimizing cancer immunotherapy may ultimately require not only better drugs but also a clearer definition and, potentially, better modulation of host immune readiness itself.
